# Market viability: a neglected concept in implementation science

**DOI:** 10.1186/s13012-021-01168-2

**Published:** 2021-11-20

**Authors:** Enola K. Proctor, Emre Toker, Rachel Tabak, Virginia R. McKay, Cole Hooley, Bradley Evanoff

**Affiliations:** 1 Present Address: Brown School, Washington University in St. Louis, One Brookings Drive, Saint Louis, MO 63130 USA; 2 Washington University Medical School in St. Louis and the University of Arizona, 1110 E. Campus Drive, P.O. Box 210033, Tucson, AZ USA 85721-0033; 3 School of Social Work, Brigham Young University, 2166 JFSB, Provo, UT 84602 USA; 4 Division of General Medical Sciences, School of Medicine, Washington University in St. Louis, 660 S. Euclid Drive, St. Louis, MO 63110 USA

**Keywords:** Implementation science, Demand, Markets, Entrepreneurship, Adoption, Sustainment, Scalability

## Abstract

This debate paper asserts that implementation science needs to incorporate a key concept from entrepreneurship—market demand—and demonstrates how assessing an innovation’s potential market viability might advance the pace and success of innovation adoption and sustainment. We describe key concepts, language distinctions, and questions that entrepreneurs pose to implementation scientists—many of which implementation scientists appear ill-equipped to answer. The paper concludes with recommendations about how concepts from entrepreneurship, notably market viability assessment, can enhance the translation of research discoveries into real-world adoption, sustained use, and population health benefits. The paper further proposes activities that can advance implementation science’s capacity to draw from the field of entrepreneurship, along with the data foundations required to assess and cultivate market demand.

Contributions to the literature

- This article introduces and explains the relevance of market viability, a new concept in implementation science.
- This article articulates similarities and differences between entrepreneurship and implementation science, and how these disciplines complement each other and advance their common goal of impact.
- This article highlights specific concepts and methods from entrepreneurship that could strengthen implementation practice and implementation science.
- This article responds to burgeoning calls to include assessments of outcomes such as cost within implementation science by highlighting market viability assessment as a candidate method.

## Introduction

Too often, questions such as, “Who will adopt interventions? “Why and how much are they willing to pay?” and “What market forces are required to sustain an intervention?” are afterthoughts in the process of intervention development. Researchers often drive the implementation of new interventions after they have developed and tested those interventions through grant-supported research. Purveyor organizations [1] and quality improvement initiatives push for adoption, sustainment, and scale-up of proven interventions, often with too little attention to demand. Whether for innovation adoption or increased use of new and better evidence-based treatments, implementation has emphasized “push out” by intervention developers more than “pull” from intervention users. All implementation requires buy-in; someone must pay if any new discovery or improvement to care is to be adopted, sustained, and scaled. Innovations must be fitted to market realities, whether that market be commercial, health system, third party payment, or fee for service. Market forces are an underemphasized concept in implementation science, as are skills for assessing and cultivating market demand.

This debate article argues that implementation science needs to more fully leverage perspectives from entrepreneurship and particularly the concept of market viability. Implementation science and entrepreneurship share a common goal, the adoption, and scale-up of innovations. These two fields differ widely in many ways—ways that are explored in this manuscript, yet entrepreneurship perspectives can benefit implementation science, practice, and training.

## Background

### Implementation science

For more than a decade, the implementation science literature has reflected the importance of acceptability, feasibility, and cost as key implementation outcomes, bearing on the uptake of innovative practices [2–4]. Further, this literature has identified treatment providers, purveyors, administrators, and payers as key stakeholders to be engaged in implementation efforts [1, 4, 5]. With few exceptions [5], too little research has addressed demand and cost considerations in adoption decisions [6–8]. Kreuter and colleagues [9] argue that marketing and distribution efforts in health and public health are “unassigned, underemphasized, and/or underfunded.” Most cost analyses of implementation focus on intervention costs, are conducted retrospectively [10, 11], and thus do not capture the potential of up-front market analysis for facilitating implementation decisions.

In implementation science, the discoveries are typically policies, procedures, protocols, interventions, care delivery pathways (guidelines), team approaches, and checklists to ensure that proven discoveries are delivered in real-world care. Evidence of an innovation’s effectiveness is a precondition for implementation, toward which a variety of strategies such as training, infrastructure redesign, protocols and checklists, and data feedback are employed to increase its adoption, sustainment, and scale-up [11]. Yet, persistent quality gaps reflect the fact that evidence alone is insufficient for implementation.

Growing evidence points to the need for researchers to consider a quality improvement initiative or an innovation’s “implementability” while developing it, specifically anticipating its potential for adoption, sustainment, and scale-up [12–16]. Implementation science has responded by embracing stakeholder engagement, considering inner- and outer-setting contexts, and using hybrid trials and user-center designs—all components of “designing for dissemination” [17–19].

Yet implementation science’s traditional skill sets seem inadequate to overcome persistent and formidable sustainment challenges. Recent literature encourages implementation researchers to get outside the “research bubble” to partner with business, the corporate sector, and healthcare payers [12, 20]. Specifically, it cites a need for expanded competencies—for understanding the business perspective on healthcare and learning how to “apply an entrepreneurial approach to spread and scale” [12]. Spread, scale, and impact are thwarted without the ability to market innovations.

### Entrepreneurship

The primary objectives of entrepreneurship are the creation, delivery, and extraction of the value of monetized products, services, or processes. Private capital has a key role in enabling the translation of basic science into financially sustainable and high social impact enterprises. According to the Angel Capital Association, in 2013, 298,000 angel investors invested $24.8 billion in about 71,000 early-stage startup companies, with pharma, biotech, and healthcare comprising 21% of angel investments in 2015 [21]. This role has become increasingly important with the past decade’s decline in the National Institutes of Health (NIH) funding [22].

Entrepreneurship involves the presence of lucrative opportunities and enterprising individuals [23]. The field can be defined as “the scholarly examination of how, by whom, and with what effects opportunities to create future goods and services are discovered, evaluated, and exploited” [24, 25]. Conceptualizing entrepreneurship as a method can help push its uses beyond technology commercialization and economic development and put it to work to build social innovations that make a positive difference in human health.

The key question entrepreneurs first ask is, “if we were to build this, would they buy?” (and if so, at what price and in what quantities?). To entrepreneurs, acceptability is understood as “willingness and ability to pay a certain price that enables a sustainable and scalable enterprise,” with market forces providing ultimate validation of innovation success [26]. Entrepreneurs recognize that to achieve long-term financial sustainability, the adequate value must be extracted (in the form of profits) to maintain the production and distribution of the innovation, and to enable the development of other related innovations. Entrepreneurs use such strategies as trialing, conducting market viability assessments, prototyping, estimating costs, and leveraging market pull forces. Entrepreneurship offers a heightened sense of urgency to deliver important solutions to the patient/marketplace. The objective is to translate a new “discovery /knowledge” to a final commercial product and to define discrete, value-generating milestones that serve as key decision points for garnering support (including financial) for subsequent steps. Prioritization of value is critical in an environment where the number of proposals for translational support greatly exceeds the available funding.

While commercialization may represent a small proportion of investments supporting health innovation, private sources of funding could be leveraged more. The National Institutes of Health’s Small Business Innovation Research (SBIR) and Small Business Technology Transfer (STTR) encourage partnerships between small businesses and non-profit research institutions, typically university-based health research programs [27]. A key objective is to translate promising technologies to the private sector and enable life-saving innovations to reach consumer markets. These “technologies” include digital health delivery platforms including patient data submitted to electronic health records via Apps or sensors, video-supported provider training programs, and patient-facing implementation strategies for behavior change. The SBIR and STTR programs invest over one billion dollars into health and life science companies that create innovative technologies aligned with NIH’s mission to improve health and save lives [27]. Implementation science seems well poised to leverage such programs, providing that it leverages demand and market forces, be they consumer, private, public, or commercial payers.

#### Implementation science and entrepreneurship

This debate paper reflects our team’s recognition that implementation science and entrepreneurship share a common goal: improving the translation and spread of new discoveries. Three propositions form the crux of this debate article, built upon foundational work in the field as cited throughout, and generating implications for the field of implementation science.

#### Proposition one: communication is challenging between implementation scientists and entrepreneurs

Each discipline has a distinct language. Unique terms are meaningful to those within a discipline but jargon typically serves as barriers to communication with those outside the discipline. The concepts of one discipline often seem foreign at best and off-putting or irrelevant at worst.

Communication between implementation science and entrepreneurship is a pre-requisite to learning and working together. In many NIH-supported Clinical and Translational Science Award programs—including that at Washington University—investigators come together across disciplines and stages of translational science in meetings and work groups. In one such event, our CTSA program employed an IDEABounce® meeting to explore the potential of pilot projects for uptake and rollout for broader application [28]. Two schools of medicine researchers who believed their work could benefit from feedback about commercial or tech support were invited to pitch, or “bounce an idea off,” a group of entrepreneurs—investors, members of start-up companies, or tech transfer groups. Researchers described the health-related problem, discovery or innovation, and opportunities for adoption and implementation. Each researcher had worked with the ICTS Dissemination and Implementation Research Core, expressing interest in how they could accelerate the uptake of the innovation and study its implementation. After the initial presentation, researchers and entrepreneurs gave feedback and asked questions of the researcher. A member of our team (a medical anthropologist) observed the meeting to observe the transdisciplinary interaction and to capture the terminology used by participants.

We observed several key communication challenges, reflected in vastly different terminology. When describing their innovations (interventions, protocols, training for new surgical techniques), the researchers emphasized the processes of development and testing, the evidence for effectiveness, service system contexts, details about research designs and analysis, potential implementation strategies, and expected outcomes. Entrepreneurs spoke about patents, scale, performance metrics organized by industry verticals, techniques for forecasting financial performance of start-ups, key benchmark performance data, start-ups, and risk/reward assessment analysis by hospital systems, digital tech companies, and insurers. The IdeaBounce meetings revealed that language differences pose a fundamental challenge to the scientific synergy between these two fields. As a result, our team asserts that implementation science could benefit from greater familiarity with the terminology around market forces, risk, reward, and return on investment analysis, and investment potential and pitch.

#### Proposition two: entrepreneurs are keenly focused on market forces

The IdeaBounce meetings further revealed differences in the information assumed to be necessary for innovation adoption and roll-out. As they listened to the “pitch” by the researchers, entrepreneurs posed questions about the market demand. They asked implementation researchers such questions as: How many people could benefit from your innovation? How great is the demand for the innovation? From whom? Who would be willing to pay for the innovation and why? What is the basis for assuming that an intervention can be profitable to potential investors? How will your innovation be sustained overtime?

For health implementation researchers, some of these questions are answered more easily than others. Most researchers can answer questions about the problem their innovation seeks to solve, the form and elements of the new treatment innovations they seek to advance, their roll-out plans, and the stakeholders and team members involved, given their consistency with the field’s foundational principles [29] and key elements of grant writing [30]. However, many of the questions about market demand, payment source options, actual costs to payers, thresholds for return on investment, and long-term sustainment plans proved extremely challenging for intervention developers and implementation researchers. Questions about who would pay, and how much they would pay, to support innovation adoption seem to stump most implementation researchers.

Some investment is typically needed for implementation. Accordingly, we argue that intervention and implementation researchers need to be better equipped for market viability assessment. Minimally, they need to be able to make data-based projections about demand and market for innovations, estimate their benefit, know who would or should pay, and forecast sustainment and scale-up challenges. If implementation researchers do not acquire these skills themselves, they need comfort and skills in partnering with entrepreneurs, payers, or investors who bring those skills.

To facilitate “designing for dissemination,” we propose a Market Viability Assessment Guide to help intervention developers, implementation researchers, and practice implementers anticipate an innovation’s adoption, sustainment, and spread. This guide draws on questions posed by entrepreneurs during the IdeaBounce and our team’s experience. Table 1 lists the questions in the guide.

**Table 1:** Market viability assessment guide

Topic	Questions from an entrepreneurial perspective
Problem or condition	What medical or public health or social problem does your innovation address? How many people are afflicted with problem? How great is the public health burden or health burden of the condition your innovation addresses?
The innovation (new treatment, diagnostic procedure, protocol, device)	How ready is your innovation for adoption in the real world? What is its readiness for adoption? How strong is the empirical evidence supporting its effectiveness?
Innovation benefit	How many people could benefit from your innovation? How soon would the benefit be realized? What is the reduction in adverse outcomes from your innovation?
Adopters	Who would use your innovation (who would put it into practice)? Who or what is the adopter group? (individual providers? Hospitals? Health systems? Patients? Communities?
Market opportunity, size, and demand	What indication do you have that your innovation is wanted? Who wants it? What is the scale and level of demand?
Comparative advantage	Do other solutions to this problem exist, and how does your innovation compare to other available solutions? What are the barriers or challenges to the adoption of your innovation?
How viable is the innovation in the adopter market?	Is the product you want to implement what the market is willing to pay for? How much will your innovation cost to develop, deliver, and market to users? (Cost) Who would pay, and why? (payer)
Innovation sustainment	What is the “invest-ability” threshold? (input/return) What is the internal rate of return (IRR) potential and potential % reduction in adverse outcomes attributable to your innovation? What will make the innovation sustainable over time?
Marketing strategy	Who is the initial target audience? How much market share can you capture?

#### Proposition three: implementation researchers and entrepreneurs have different but potentially complementary priorities, values, and norms

In addition to differences in communication and priority questions, our observations suggest that implementation researchers and entrepreneurs have fundamental differences in priorities, values, and norms. One example is the emphasis on data and valuations of different data types. In implementation science, an innovation’s readiness for implementation is viewed as a primary function of the strength of evidence for its effectiveness [30–32]. Within a private enterprise, reliance on data varies. Institutional venture capital firms deploy data-driven metrics to assess new venture viability. However, the literature suggests that most early-stage start-ups assess viability subjectively based largely on experience and perceived market demand [33, 34].

Risk tolerance is another factor perceived differently. Entrepreneurs may couch risk in terms of willingness and ability to take a financial risk, while the healthcare system might focus more on preventing adverse outcomes. For angel-invested early-stage start-ups, failure rates of nearly 75% are common [35], contributing to greater comfort with failure—at least financial failure because the next venture’s success could cover financial loss. By contrast, health administrators and researchers may approach risk more warily due to financial constraints and the prevailing ethic of “do no harm” [13]. Most entrepreneurs and investors base their decisions on the assumption that potential reduction in adverse healthcare outcomes determines profitability and therefore, investability with adverse outcomes broadly defined as anything that costs the healthcare system money.

As shown in Table 2, implementation science and entrepreneurship valued different products, processes of development, and perceived return on investment. Such differences in norms and cultures likely affect the ease of collaboration. Researchers vary in their comfort with technology supports and business/entrepreneurial partnerships: many intervention developers and implementation researchers express distrust for working with potential investors from commercial enterprise: “I don’t’ want to share my idea with investors because I’m afraid they might steal my idea.” More fundamentally, business, cost, and market analysis have been viewed as irrelevant to implementation science—as somebody else’s concern.

**Table 2:** Priorities, norms, and values of implementation science and entrepreneurship

Readiness	Implementation science	Entrepreneurship
	Evidence	Market demand
Return on investment	Quality of care Public health reach Health status	Profitability Market share Market size (size of customer base)
Process	Sequential Incremental	Iterative Big leaps: “think big, start small” Risk/reward profile
Risk tolerance	Low	High
Products	Grants Publications	Services Products

#### Implications for implementation science

Research translation will be accelerated and strengthened when implementation science can benefit from entrepreneurship. Implementation science needs to leverage several key lessons from the entrepreneurial world [36, 37], particularly its emphasis on market demand and return. We propose several directions for leveraging these benefits for the field of implementation science.

##### Increase emphasis on adoption markets

Adoption is a widely used term and a key concept in implementation science, constituting a key goal of implementation processes and hence a key implementation outcome [4, 38]. Yet, implementation science evidences only sparse attention to adoption *markets*. Intervention developers and implementation researchers need to appreciate not only the importance of their discoveries but also the potential demand for them. Implementers need to know who the potential adopters are, who are the key decision makers in an adoption, how the adoption context affects receptivity to and demand for new interventions, and how they compare to available or potentially available alternatives. Innovations may be less acceptable in already crowded markets [39].

##### Increase ability to communicate with payers and how to communicate in market-relevant terms

The individuals and groups who make final decisions about adopting new interventions, particularly those making investment decisions for provider organizations, consider concepts that rarely appear in the implementation science literature, such as those reflected in Table 2 above. Adopters consider the return on investment, investment rates of return (IRR) potential and time to investment return, and reduction in adverse outcomes attributable to the intervention and its implementation. Implementation science training should incorporate fluency in these terms as well as an understanding of various risk calculations [13, 20].

##### Forge working relationships with entrepreneurs, investors, and innovators from corporate sectors to explore market viability

Implementation scientists frequently partner with other disciplines, such as communication and decision science, organizational psychology, systems engineering, medicine, psychology, anthropology, social work, and increasingly economics. Entrepreneurship experts can provide valuable feedback on the clarity of the idea, the persuasion of presenters’ messages, and the project’s potential for advancing health. To date, published literature reflects few examples of partnering with corporate investors or entrepreneurs. However, CTSAs across the country are forging such partnerships and offering new courses and training programs in entrepreneurship, and the National Cancer Institute has launched the SPRINT program to harness business principles to accelerate research translation [10, 15, 16, 40](https://www.uab.edu/ccts/training-academy/trainings/innovation-and-entrepreneurship/i-corps). Our team’s experience confirms the benefits of such initiatives [13]. IdeaBounce events such as we convened can be particularly helpful to investigators with projects in the early stages of development, better equipping them to “design for dissemination” [17, 41]. Convening “jargon translation clinics” can enhance the ability of intervention developers and implementation researchers to communicate the comparative advantage of new discoveries and clarify terminology that may be familiar to investors and payers. Entrepreneurs can help assess market, demand, and potential commercialization of discoveries, and early assessment of feasibility and market viability may accelerate adoption and sustained implementation.

##### Prioritize development of data to support market analysis for health innovations and advance cost assessment methods

Market viability requires knowledge of implementation costs as well as the range of payers. Competitive insurance markets, formularies, and a mix of fee-for-service and bundled payment schemes make intervention costs difficult if not impossible to determine. Psychosocial interventions are particularly hard to cost, as are implementation strategies required for adoption, sustainment, and scale-up. Implementation scientists need to be able to identify, if not to calculate, costs associated with developing an innovation, along with costs and savings to healthcare systems from using an innovation. Presently, the data needed are under-developed, imprecise, incomplete, and often obscured or hidden within insurance costs and preferred provider markets [42, 43]. Although cost concerns are associated with stakeholder reluctance to implement evidence-based interventions, and often are the most significant barrier to their implementation [44, 45], implementation science training has not sufficiently emphasized skills for costing or market analysis. Guidance on methods to cost implementation initiatives are a growing and welcome addition to the literature [2, 46, 47], particularly those that include long-term benefit [42] and include downstream costs that are highly relevant to decision makers [48].

Entrepreneurship relies on key performance metrics, based on historical data generated by prior new ventures. However, such data for health is extremely fragmented and has not yet been accumulated, curated, and organized in a national database. Having access to historical key performance metrics collected from other similar innovation rollouts would be extremely helpful to stakeholders in quantifying risk/reward profile assessments of innovations. For example, a hospital’s data on “time required to train in new procedures” can inform their assessment of the value in investing in new technologies, based on the “time to investment return.”

##### Feasibility analysis clinics

Implementation research cores, training programs, and labs can convene “clinics” or workshops to bring entrepreneurs and researchers together. Insurance officials, healthcare chief financial officers, and experts in social and commercial entrepreneurship can help intervention developers and implementation researchers identify business-driven tools for evaluating the need and assessing demand in real-world healthcare. Their expertise can help dissemination and implementation researchers develop skills for assessing feasibility and market viability [49] and creating “business plans” for adoption, scale-up, and sustainment for improvements to care.

##### Expand research toolkits by including resources from each field—implementation science and entrepreneurship—and provide investigators opportunities to use them

Within each field—entrepreneurship and implementation science—a growing number of toolkits provide resources to support research and commercial rollout [50]. Existing toolkits should be made available across disciplinary boundaries. Moreover, toolkits should be developed that provide market viability assessment tools for the implementation science field.

Activities such as these can help accelerate the translation of research findings by equipping the research workforce with core competencies not only in their own disciplines but in complementary areas—in this case, implementation science and entrepreneurship, and also to effectively communicate and collaborate as members of multidisciplinary teams.

## Summary

Implementation science and entrepreneurship share the goal of moving discoveries from the lab to the bedside. Yet, in most university settings, their paths barely cross. We need to better understand the ways implementation science can benefit from entrepreneurial conceptualizations and tools, including those for marketing and design. A primary lesson from entrepreneurship is the importance of understanding and effectively leveraging market forces for implementation. Push-out is important but insufficient for implementation success. Cultivating demand is also important, as leveraging market forces can facilitate adoption and sustainment. Entrepreneurship can provide a network of expertise outside the academic community to bring in diverse, expert, and critical feedback to evaluate the feasibility of proposals and aid in the design of viable projects, consistent with implementation science’s high value on stakeholder engagement. A 2014 Implementation Science editorial stated, “The question is how economic evaluation can most efficiently be incorporated into implementation decisions, not whether it should” [8]. We extend this position by stating, “The question is how market analysis can best inform and facilitate implementation processes, not whether it should.”

## Data Availability

Notes from our team’s work in the Implementation Science-Entrepreneurship Unit, specifically IdeaBounce meetings and implementation researchers’ responses to questions posed by entrepreneurs, are available from the corresponding author upon request.

## Abbreviations

CTSA: Clinical and Translational Science Awards Program
ICTS: The Washington University in St. Louis Institute for Clinical and Translational Science (ICTS)
ISE: Implementation Science-Entrepreneurship Unit
NIH: National Institutes of Health
